# Effects of early enteral nutrition on the gastrointestinal motility and intestinal mucosal barrier of patients with burn-induced invasive fungal infection

**DOI:** 10.12669/pjms.323.9717

**Published:** 2016

**Authors:** Yu Zhang, Fang Gu, Fengxian Wang, Yuanda Zhang

**Affiliations:** 1 Yu Zhang, Baoding Children’s Hospital, Baoding 071000, Hebei Province, China; 2 Fang Gu, Baoding Children’s Hospital, Baoding 071000, Hebei Province, China; 3 Fengxian Wang, Baoding Children’s Hospital, Baoding 071000, Hebei Province, China; 4 Yuanda Zhang, Baoding Children’s Hospital, Baoding 071000, Hebei Province, China

**Keywords:** Arginine, Burn, Enteral nutrition, Fungal infection, Gastrointestinal motility, Intestinal mucosal barrier

## Abstract

**Objective:**

To evaluate the effects of early enteral nutrition on the gastrointestinal motility and intestinal mucosal barrier of patients with burn-induced invasive fungal infection.

**Methods:**

A total of 120 patients with burn-induced invasive fungal infection were randomly divided into an early enteral nutrition (EN) group and a parenteral nutrition (PN) group (n=60). The patients were given nutritional support intervention for 14 days, and the expression levels of serum transferrin, albumin, total protein, endotoxin, D-lactic acid and inflammatory cytokines were detected on the 1st, 7th and 14th days respectively.

**Results:**

As the treatment progressed, the levels of serum transferrin, albumin and total protein of the EN group were significantly higher than those of the PN group (P<0.05), while the levels of serum endotoxin and D-lactic acid of the form group were significantly lower (P<0.05). After treatment, the expression levels of IL-6 and TNF-α were decreased in the EN group, which were significantly different from those of the PN group (P<0.05). During treatment, the incidence rates of complications such as abdominal distension, diarrhea, sepsis, nausea, vomiting and gastric retention were similar. The mean healing time of wound surface was 9.34±0.78 days in the EN group and 12.46±2.19 days in the PN group, i.e. such time of the former was significantly shorter than that of the latter (P<0.05).

**Conclusion:**

Treating patients having burn-induced invasive fungal infection by early enteral nutrition support with arginine can safely alleviate malnutrition and stress reaction, strengthen cellular immune function and promote wound healing, thereby facilitating the recovery of gastrointestinal motility and the function of intestinal mucosal barrier.

## INTRODUCTION

Generally, patients with severe burn (a total burn area of over 30%) are in critical conditions. Sharp decline in immune function, necrotic tissues and broad-spectrum antibiotics create conditions for fungal infections.^1^,^2^ Due to acute, severe, intense and persistent excessive stress responses, it is initially manifested as low metabolism which, however, soon accelerates, causing violent reactions in nervous, endocrine, metabolic and other systems. Meanwhile, energy substances undergo increased decomposition and decreased synthesis, and utilization of glucose in peripheral tissues is limited, so enough calories and proteins must be provided to maintain high-energy metabolism and to reduce the serious consequences brought about by high catabolism.^3^-^5^ Besides, with increasing antibiotics use, fungal infections and drug resistance have aggravated.^6^

Upon severe burn, nutritional support crucial, so rational nutrition intervention can reduce infectious complications and promote the recovery of patients. Enteral nutrition in combination with special nutrients can significantly reduce the duration of systemic inflammatory response syndrome and the incidence of multiple organ failure of burn patients.^7^ Arginine can improve immune function, reduce catabolism, promote protein synthesis and protect gastrointestinal mucosa, with increasingly evident effects on clinical nutrition therapy.^8^ In the meantime, arginine can relieve ischemia-reperfusion injury through gastrointestinal supplementation to help protect the function of intestinal mucosal barrier. In addition, arginine metabolism is able to balance immune response, and polyamines are generated by the arginase pathway to enhance cell activity, to regulate immunity and macrophages, to inhibit platelet adhesion and to participate in sterilization.^9^ Current studies have indicated that there are interactions between inflammatory response and burn coagulation, and severe burn patients are often accompanied by inflammation deficits.^10^ To this end, we evaluated the effects of early enteral nutrition on the gastrointestinal motility and intestinal mucosal barrier of patients with burn-induced invasive fungal infection.

## METHODS

### Subjects

A total of 120 patients with burn-induced invasive fungal infections, who were received in emergency in our hospital between August 2011 and December 2013, were selected. This study was approved by the ethics committee of our hospital, and written consent has been obtained from all patients.

### Inclusion criteria

In accordance with the diagnostic criteria of burn-induced invasive fungal infection; wounds were distributed in face, trunk and limbs; amino acids, high-fat milk or high-sugar nutrients were not given intravenously; all patients were treated by cefoperazone/sulbactam, imipenem and other broad-spectrum antibiotics; the expected lifetime was longer than 6 months; 18-70 years old patients of either gender; adaptable to nutritional therapy.

### Exclusion criteria

Patients with diabetes, cancers and HIV-positive results; patients who had used non-topical nutritional supplements containing zinc, vitamin E or arginine within one month prior to screening; with hepatic, renal or cardiac dysfunction; with history of drug abuse or alcoholism.

The patients included 66 males and 54 females, aged between 22 and 68 years old (average: 45.34±1.97). The total burn areas ranged from 31% to 78%, with an average of 45.98±2.11%. The average height was 166.44±5.63 cm and the average weight was 58.30±5.43 kg.

### Reasons for burn

25 cases were of blast injury, 15 cases of chemical burn, 15 cases of steam scalding and 65 cases of fire burn. The times from burns to fungal infection ranged from 6 h to 24 h after injury, with an average of 13.34±2.89 h.

### Sites of infection

In 78 cases on the wound surface, 22 cases in the respiratory tract, 12 cases in the throat, 6 cases in the urinary system and 2 cases in blood. There were 68 cases of deep vein catheterization, 28 cases of ventilator treatment and 44 cases of routine tracheotomy, all of whom had urinary catheter retention.

The patients were randomly divided into an early enteral nutrition (EN) group and a parenteral nutrition (PN) group (n=60). There were no statistically significant differences in baseline clinical data such as gender, age, area of burn, causes, occurrence time of fungal infection, site of infection or clinical diagnosis and treatment between the two groups (P>0.05).

### Treatment methods

After fungal infection, broad-spectrum antibiotics were discontinued immediately. Necrotic tissues were removed, and wound surfaces were dressed with fluconazole powders and washed with 2% iodine tincture or iodine, once a day. At the same time, the patients received itraconazole injection, 200 mg for the first day (twice a day) and 200 mg/d afterwards. With respect to nutrition intervention, both groups were given standard enteral nutrition Nutrison Fibre [Nutricia Pharmaceutical (Wuxi) Co., Ltd., 20111209]. On this basis, the EN group was given arginine enteral nutrient solution Cubison (Nutricia, Netherlands, 20110934, containing 8.5 g/L arginine). With diet restricted, all patients were injected with 24 IU of insulin daily by micropump. Both groups took 1000 kcal of enteral nutrition agent within 48 hour after inclusion, and the remaining energy was supplemented through food intake. Daily calorie supply was estimated according to the following formula: (kcal/d) = 1000 × body surface area (m^2^) + 25 × burn area (%). The intervention of nutritional support of the two groups both lasted 14 days.

### Observation indices

### Changes in serum proteins

After different nutritional therapies, 3 ml of fasting blood was drawn on the morning of 7th and 14th days. The levels of serum total protein, albumin and transferrin were detected by Hitachi 7600 automatic biochemical analyzer.

### Levels of serum endotoxin and D-lactic acid

The levels of serum endotoxin and D-lactic acid were detected on 7th and 14th days after surgery. Serum endotoxin was detected using the modified azo chromogenic method,^11^ and serum D-lactic acid was determined by modified enzyme spectrophotometry.^12^

###  Changes in inflammatory cytokines

Fasting venous blood (3 ml) was drawn at the same time point to detect the expressions of inflammatory cytokines [interleukin-6 (IL-6) and tumor necrosis factor-α (TNF-α)] using enzyme-linked immunosorbent assay. All the detections were performed in strict accordance with the instructions.

### Prognosis

The healing time of wound surface was observed. The criteria for healing were fresh wound, no secretions or obviously mitigated inflammatory responses.

###  Complications

The complications of all patients were observed during the treatment, including abdominal distension, diarrhea, sepsis, nausea, vomiting, gastric retention, etc.

### Statistical analysis

Statistical analysis was performed using Wstata software. The data were expressed as mean±standard deviation. The categorical data were compared by inter-group t test, and the numerical data were compared by chi-square test. P<0.05 was considered statistically significant.

## RESULTS

### Changes in serum proteins

With progress of the treatment, the levels of serum transferrin, albumin and total protein of the two groups were significantly increased (P<0.05). The levels of the EN group were significantly higher than those of the PN group after treatment (P<0.05) (Table-I).

**Table-I:** Changes in serum proteins before and after treatment (g/L, x±s).

*Group*	*Case No. (n)*	*Transferrin*	*TP*	*Albumin*
PN group -				
0 day	60	1.72±0.45	53.44±8.34	24.98±4.54
7th day	60	2.21±0.76	59.38±8.98	29.64±6.49
14th day	60	2.46±0.97	64.76±11.23	29.87±6.44
EN group -				
0 day	60	1.73±0.45	51.33±8.48	26.18±4.59
7th day	60	2.36±0.82#	63.76±9.21#	33.34±5.31#
14th day	60	2.86±0.76#	66.54±6.87#	35.87±4.98#

Compared with the PN group,

# P<0.05.

### Changes in levels of serum endotoxin and D-lactic acid

The levels of serum endotoxin and D-lactic acid of the PN group were significantly lower than those of the PN group (P<0.05) (Table-II).

**Table-II:** Changes in levels of serum endotoxin and D-lactic acid (x±s).

*Group*	*Case No. (n)*	*Endotoxin (EU/ml)*	*D-lactic acid (mg/L)*
PN group -0 day	60	0.37±0.04	11.35±2.03
7th day	60	0.55±0.02	17.72±3.51
14th day	60	0.67±0.04	17.97±2.73
EN group -0 day	60	0.35±0.01	11.71±5.01
7th day	60	0.41±0.07#	15.13±2.29#
14th day	60	0.46±0.04#	13.12±2.52#

Compared with the PN group,

# P<0.05.

### Changes in inflammatory cytokines

The levels of IL-6 and TNF-α of the EN group were decreased after treatment, being significantly different from those of the PN group (P<0.05) (Table-III).

**Table-III:** Changes in inflammatory cytokines before and after treatment (ng/ml, x±s).

*Group*	*Case No. (n)*	*IL-6*	*TNF-α*
PN group -0 day	60	288.36±14.98	8.64±1.55
7th day	60	182.44±33.92	6.43±1.09
14th day	60	120.97±22.34	5.01±0.98
EN group -0 day	60	289.65±20.37	8.66±1.34
7th day	60	153.66±31.52#	4.08±1.32#
14th day	60	95.67±18.54#	3.65±0.98#

Compared with the PN group,

# P<0.05

### Complications

There were no significant differences between the two groups as regards complications during treatment, such as abdominal distention, diarrhea, sepsis, nausea, vomiting and gastric retention (Table-IV).

**Table-IV:** Incidence rates of complications during treatment (n).

*Group*	*Case Number*	*Abdominal Distension*	*Diarrhea*	*Septicemia*	*Nausea and Vomiting*	*Gastric Retention*
PN group	60	4(6.7%)	3(5.0%)	2(3.3%)	6(10.0%)	4(6.7%)
EN group	60	3(5.0%)	2(3.3%)	2(3.3%)	5(8.3%)	4(6.7%)
X2		0.076	0.081	0.000	0.041	0.000
P		>0.05	>0.05	>0.05	>0.05	>0.05

### Healing time

The average healing time of wound surface was 9.34±0.78 days in the EN group and 12.46±2.19 days in the PN group, i.e. such time of the former was significantly shorter than that of the latter (P <0.05) (Table-V).

**Table-V:** Average healing time (d, x±s).

*Group*	*Case Number (n)*	*Healing Time*
EN group	60	9.34±0.78
PN group	60	12.46±2.19
t		9.112
P		<0.05

## DISCUSSION

Reasonable nutritional support and metabolic intervention play key roles in promoting burn wound healing. Current studies have indicated that early enteral nutrition therapy is safe and effective, which can improve metabolic reactions and the entire clinical course, thus shortening the healing time. In recent years, arginine has attracted wide attention due to its beneficial effects on clinical nutrition therapy.^13^ Under normal conditions the amount of arginine can meet daily needs. However, upon burns, human body cannot maintain a positive nitrogen balance or normal physiological functions, while exogenous arginine can alleviate immune hypofunction and increase protein synthesis after burn traumas.^14^

In the nutrition indices of burn patients, both transferrin and albumin are acute phase proteins synthesized in the liver, and the metabolism of proteins can be sensitively reflected in patients without metabolic disorders. Severe malnutrition may cause atrophy of immune organs, promote inflammation and translocate intestinal bacteria.^15^ In this study, with progress of treatment, the levels of serum transferrin, albumin and total protein were all significantly elevated in both groups (P<0.05), and those of the EN group were significantly higher after treatment (P<0.05). Therefore, enteral nutrition support using arginine can improve the nutritional status of patients with severe burns.

Physiological damage of intestinal barrier function is a fundamental pathophysiological factor causing bacterial translocation during which intestinal bacteria invade intestinal tissues through intestinal epithelial cells. Bacterial toxins can also enter the blood circulation through abnormal epithelial barrier. Endotoxemia and function failure of intestinal mucosal barrier may lead to exacerbation of patients with hepatic cirrhosis. D-lactic acid is produced by inherent bacteria in the gastrointestinal tract. As mammals do not have enzymatic systems capable of rapid metabolism, the D-lactic acid level in blood may timely reflect changes in intestinal mucosa permeability.^16^ When immunosuppression is caused by burns and severe infection, the effects of nutritional support are limited, thus needing nutrients that can regulate immunity and enhance immune function. Reasonable doses of arginine can effectively promote cellular immune function and increase the resistance of human body to infection through improving the phagocytic capacity of macrophages. Animal experiments have shown that feeding or intravenous supplementation of arginine significantly reduced the atrophy of thymus and improved cellular immune function.^17^

Severe burn patients can produce large amounts of proinflammatory cytokines such as TNF-α and IL-6, while exogenous supplementation of arginine can reduce the gene expressions of TNF-α and IL-6 on the basis of early enteral nutrition to further improve the synthesis of liver albumin. TNF-α is a major cytokine activated by acute phase responses after burn. The release of TNF-α and a series of cytokine such as IL-6 induced by it is one of the main reasons for multiple organ failure after burn.^18^ As the treatment progressed herein, the levels of serum IL-6 and TNF-α were significantly reduced in both groups compared with those before treatment (P<0.05). After treatment, the serum IL-6 and TNF-α levels of the EN group were significantly lower than those of the PN group, between which the differences were statistically significant (P<0.05). Accordingly, enteral nutrition support using arginine may regulate the acute phase response after burn by inhibiting the release of TIL-6 and TNF-α, so as to promote the synthesis of visceral protein in liver cells. Enteral nutrition helps maintain the structural and functional integrities of the intestinal mucosa, and the irritation of food on intestinal mucous membrane is conducive to promoting the secretion of gastrointestinal hormones, gallbladder contractibility and gastrointestinal peristalsis as well as protecting the intestinal barrier function.^19^ In this study, there were no significant differences between both groups during treatment in the incidence rates of complications such as abdominal distension, diarrhea, sepsis, nausea, vomiting and gastric retention. In addition, the mean healing time was 9.34±0.78 days in the EN group and 12.46±2.19 days in the PN group, of which the former was significantly shorter than the latter (P<0.05). During the nutritional support using arginine, we used a constant input by micro-feed pump at fixed quantity and time, which gradually recovered the intestinal function, strengthened the tolerance, reduced the incidence of gastrointestinal complications, and eventually contributed to wound healing.

In summary, early enteral nutrition using arginine in patients with burn-induced invasive fungal infection can safely alleviate malnutrition and stress reaction, strengthen cellular immune function and facilitate wound healing, thereby promoting gastrointestinal motility and the recovery of mucosal barrier function.
